# Prevention of Lower Urinary Tract Symptoms (PLUS) Research Consortium—Update for Society of Urodynamics, Female Pelvic Medicine and Urogenital Reconstruction (SUFU)

**DOI:** 10.1002/nau.70230

**Published:** 2026-02-04

**Authors:** Ariana L. Smith, Siobhan Sutcliffe, the Prevention of Lower Urinary Tract Symptoms (PLUS) Research Consortium, Linda Brubaker, Colleen Fitzgerald, Marian Acevedo‐Alvarez, Cecilia T. Hardacker, Jeni, Hebert‐Beirne, Elizabeth Mueller, Elise Levin, James W. Griffith, Kimberly Sue Kenton, Margaret Mueller, Melissa Marquez, Melissa Simon, Oluwateniola Brown, Julia Geynisman‐Tan, Alayne D. Markland, Camille P. Vaughan, Kathryn L. Burgio, Cora E. Lewis, Gerald McGwin, Beverly Rosa Williams, Sara Elgayar, Emily S. Lukacz, D. Yvette LaCoursiere, Jesse Nodora, Dulce Rodriguez‐Ponciano, Joseline Sanchez, Kyle Herrala, Lisa Kane Low, Janis M. Miller, Abby Smith, Eliza Wilson‐Powers, Gerald McGwin, Kyle D. Rudser, Sonya S. Brady, Cynthia S. Fok, Bernard L. Harlow, Peter, Scal, Todd Rockwood, Chloe Falke, Elise Steichen, Lucas Zhang, Sara Putnam, Amy Claussen, Vanika Chary, Diane K. Newman, Ariana L. Smith, Amanda Berry, Andrea Bilger, Terri H. Lipman, Heather Klusaritz, Ann E. Stapleton, Jean F. Wyman, Kristine Talley, Katlin Nuscis, Siobhan Sutcliffe, Aimee S. James, Jerry L. Lowder, Melanie R. Meister, Amy Ayala, Julia Maki, Ratna Pakpahan, Leslie M. Rickey, Deepa R. Camenga, Shayna D. Cunningham, Linda Brubaker, Jenna Norton

**Affiliations:** ^1^ Division of Urology Perelman School of Medicine at the University of Pennsylvania Philadelphia Pennsylvania USA; ^2^ Division of Public Health Sciences, Department of Surgery Washington University in St. Louis St. Louis Missouri USA; ^3^ Loyola University Chicago Maywood IL; ^4^ University of Chicago Chicago IL; ^5^ Northwestern University Chicago IL; ^6^ University of Alabama at Birmingham Birmingham AL; ^7^ University of California San Diego La Jolla CA; ^8^ University of Michigan Ann Arbor MI; ^9^ University of Minnesota (Scientific and Data Coordinating Center) Minneapolis MN; ^10^ University of Pennsylvania Philadelphia PA; ^11^ Washington University in St. Louis Saint Louis MO; ^12^ Yale University New Haven CT; ^13^ NIH Program Office: National Institute of Diabetes and Digestive and Kidney Diseases, Division of Kidney, Urologic, and Hematologic Diseases Bethesda MD

## Abstract

The Prevention of Lower Urinary Tract Symptoms (PLUS) Research Consortium, supported by the National Institute of Diabetes and Digestive and Kidney Diseases (NIDDK), has had a successful and productive ten years, accomplishing several major achievements, including over 65 peer‐reviewed publications. This multicenter, transdisciplinary Consortium developed the concept of bladder health and studied it systematically in RISE FOR HEALTH (RISE). The resources developed and the data generated will be made available for use through the NIDDK repository, providing the content for many more accomplishments in the field of bladder health.

**Clinical Trial Registration:** NCT05365971

## 1 The PLUS Research Consortium

The Prevention of Lower Urinary Tract Symptoms (PLUS) Research Consortium received ten years of funding from the National Institute of Diabetes and Digestive and Kidney Diseases (NIDDK) to understand bladder health among women and girls. At the 2025 Society of Urodynamics, Female Pelvic Medicine, and Urogenital Reconstruction (SUFU) annual meeting in Palm Springs, California, the major accomplishments of the PLUS Research Consortium, including its more than 65 peer reviewed publications, were presented to share the latest understanding of bladder health and the breadth of data and research tools generated that are available to the scientific community for the next phase of study on bladder health promotion and lower urinary tract symptom (LUTS) prevention.

The PLUS Research Consortium is made up of a scientific and data coordinating center (SDCC; University of Minnesota) and eight clinical research centers (Loyola University, Northwestern University, University of Alabama, University of Michigan, University of Pennsylvania, University of California San Diego, Washington University at St. Louis, and Yale University). The Consortium was built with a vision of transdisciplinary team science. Multiple disciplines are represented in the over 50 PLUS members, including prevention scientists, measurement experts, qualitative researchers, biostatisticians, epidemiologists, urologists, urogynecologists, geriatricians, adolescent health clinicians, psychologists, physiatrists, and more.

The first task of the membership was to come together as a team of scientists and share diverse perspectives and expertise. The early work of the Consortium included developing and publishing a definition of bladder health (“A complete state of physical, mental, and social well‐being related to bladder function, and not merely the absence of lower urinary tract symptoms [LUTS]).” [1] Healthy bladder function “permits daily activities, adapts to short‐term physical and environmental stressors, and allows optimal well‐being (e.g., travel, exercise, social, occupational, or other activities).” Next, the Consortium developed and published a conceptual framework to guide bladder health etiologic research [2]. This framework integrates a life course perspective, acknowledging that different factors impact the bladder at different times in life, a social ecological perspective, recognizing that health behaviors are influenced by multiple social factors outside of the individual, and biological considerations. The Consortium also developed infrastructure for community engagement in PLUS research to promote inclusivity of populations underrepresented in research, such as sexual and gender minorities, ethnic and racial minorities, and women young and old.

## 2 A Tool to Measure Bladder Health

Before PLUS, there was no measure of bladder health, and existing LUTS measures lacked the ability to measure the full range of bladder health across the multiple ways in which the bladder impacts an individual. Gaps in knowledge and understanding were filled with one of our first studies, a qualitative study called SHARE (Study of Habits, Attitudes, Realities and Experiences), designed to hear women and girls′ voices about their bladder [3]. Thirty focus groups were conducted across seven PLUS research centers to explore experiences, perceptions, knowledge, and behaviors related to the bladder of women and girls 11 years of age and older. Several dimensions of bladder health emerged through these conversations. These fell into three main buckets: concepts around (1) holding/storing urine, (2) voiding/emptying the bladder, and (3) functional/psychosocial impacts on the bladder. We used these data to develop draft items for a new bladder health measure.

Cognitive evaluation and testing of the items making up the new measure took place as part of our next study, Clarification of Language, Evaluation, And Refinement of questions (CLEAR) [4]. This study incorporated cognitive interviews (*n* = 167) with structured probing questions to ensure content validity with each survey item stem and response option. Using an iterative process, item stems and response options were refined, and different versions of items were developed for testing using an online e‐panel (*n* = 791) prior to validation.

The CLEAR study produced our new comprehensive bladder health instrument, the 10 Bladder Health Scales and six Bladder Function Indices (BHS/BFI, Table 1) [5]. Each bladder health scale (Table 1, left column) is made up of a few questions that measure an individual′s perception of their bladder and how well it works in various situations. The six bladder function indices (Table 1, center column) measure how well the bladder performs or functions. The instrument also captures adaptive or coping behaviors related to the bladder (Table 1, right column). The total number of items is 76, producing a score of 0‐100, with 100 indicating perfect bladder health. A general perception of bladder health, or what was termed “Global Scale,” has only six items and can be used alone or in combination with the other scales and indices. The new measure was validated for use and published in 2022 [5].

**Table 1:** Components of the bladder health scales/bladder function indices (BHS/BFI).

**Bladder Health Scales**	**Bladder Function Indices**	**Adaptive/Coping Behaviors**
Global perception	Biosis/urinary tract infection (UTI)	Wearing or carrying incontinence liners, pads, or absorbent underwear
Ease of holding	Frequency	Toilet mapping
Perceived efficacy	Sensation	Staying as close to a bathroom as possible
Social/occupational	Continence	
Physical activity	Comfort	
Intimacy	Emptying	
Travel		
Emotional		**Scoring 0–100** **Higher score = Better health**
Impact on life		
Freedom from worry about the bladder		

## 3 Factors Impacting Bladder Health

**Figure 1:**
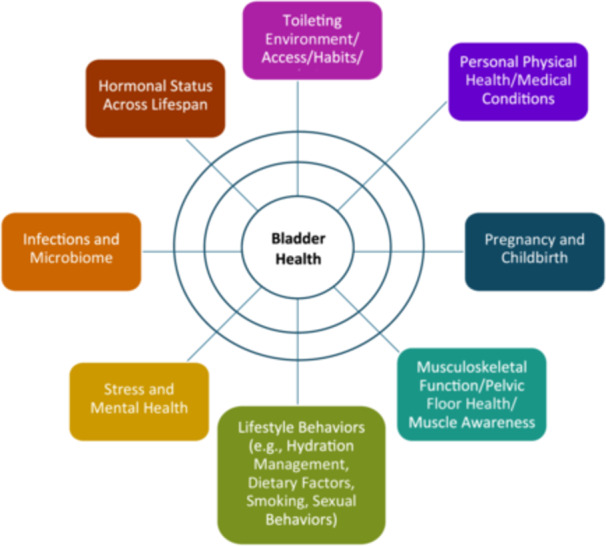
Eight broad research themes for the study of bladder health [6].

In parallel with bladder health instrument development, the Consortium developed a list of candidate bladder health risk and protective factors to study in a new cohort study, the RISE FOR HEALTH study (RISE). RISE was designed to: (1) identify factors associated with bladder health and LUTS in US women, and (2) determine the distribution of bladder health and bladder health knowledge in US women using the new measure [6]. The Consortium started by developing a comprehensive list of potential risk and protective factors using the PLUS conceptual framework. There were more than 600 factors generated that were clustered into the eight broad research themes (Figure 1): (1) Toileting Environment/Access/Habits/Techniques, (2) Personal Physical Health/Medical Conditions, (3) Pregnancy/Childbirth, (4) Musculoskeletal Function/Pelvic Floor Health/Muscle Awareness, (5) Lifestyle Behaviors (e.g., Fluid Intake, Diet, Smoking, Sexual Behaviors), (6) Stress and Mental Health, (7) Infections and Microbiome, and (8) Hormonal Status Across Lifespan. Across the 8 research themes, 27 research questions were prioritized for study in the Consortium. To answer these 27 research questions, the Consortium used rigorous psychometric principles to develop several new instruments in addition to the BHS/BFI [5].

**Figure 2:**
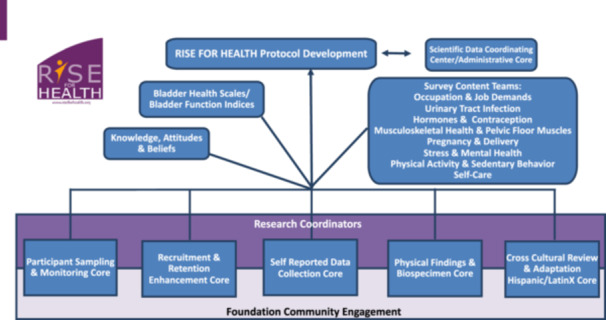
Organizational structure for the RISE FOR HEALTH study development.

Much work was also dedicated to the design of RISE to optimize its ability to address the 27 research questions and to obtain population‐based estimates of bladder health in the female community‐dwelling population. The transdisciplinary nature of the Consortium provided expertise in a variety of scientific methods (e.g., qualitative research, probability sampling, survey development, epidemiology, biostatistics, and community‐based participatory research) to facilitate the development of this large, multifaceted study. The Consortium built an organizational structure (Figure 2) to support RISE study development, valuing scientific rigor, feasibility, and inclusion. With community engagement at the foundation, cores were formed to: (1) develop participant sampling strategies (valuing broad demographic representation and feasibility), (2) optimize response and retention of participants, (3) develop survey content and streamline participants′ survey experience, (4) develop the protocols for physical examination measures and biospecimen collection (i.e., urine, vaginal, and rectal swabs), and (5) adapt and translate materials for use by Hispanic and Spanish speaking participants. Community partnerships were developed (e.g., Community Partnership Council) to formally engage community members. These groups served as advisors on a variety of study development needs, including survey content review, participant recruitment materials, and website development. The self‐reported data collection core compiled the survey for RISE, including our new bladder health instrument. Consortium content experts developed and validated additional instruments to measure novel factors such as toileting environment, bladder knowledge and beliefs, and self‐care practices. To measure covariates, existing validated measures were used when available. These survey measures formed the toolbox for RISE.

RISE was launched in 2022 using stratified random sampling to recruit a sample of participants that were ethnically and racially similar to the United States population across a wide range of ages (18–100 years). A total of 50 367 invitations were sent with a 7.9% (*n* = 3422) response rate; the mean age of the cohort was 49.8 years [7]. Data were collected via mailed or emailed surveys and in‐person assessment (*n* = 520); urine specimen collection (*n* = 435) took place in a subset of participants. An embedded pilot study (*n* = 95) produced both vaginal and rectal specimens. Baseline assessments began in May 2022 and were completed in December 2023. Mid‐year newsletters and 1‐year and 2‐year follow‐up assessments were sent to enrolled participants.

## 4 Early Findings From RISE FOR HEALTH

RISE achieved ethnically and racially representative sampling, with 15.3% of participants identifying as Hispanic; 5.9% as non‐Hispanic Asian; 12.3% non‐Hispanic Black; 62.9% non‐Hispanic White; and 2.6% multiple racial identities [8]. RISE also achieved a representative sampling of age across the adult lifespan; however, sexual and gender minorities may have been underrepresented. RISE participants were more highly educated and more likely to be insured. Due to 20% missing data on household income, it is unclear how our study participants relate to the United States population. There was an expected distribution of body mass index (BMI) and menopausal stages (pre‐, peri‐, and post‐) in RISE participants.

LUTS were measured using the Lower Urinary tract Research Network Symptom Index‐10 (LURN‐SI‐10). At least one mild LUT was present in 79.2% of participants, and 36.5% experienced at least one moderate or severe symptom [9]. These findings were similar to other population‐based surveys on LUTS [10, 11]. Urgency was the most prevalent LUT observed in more than 55% of participants, while UUI was observed in 32.3%. Bother from urinary symptoms was present in 37.5% of participants, and 37.9% had discussed concerns about urinary symptoms with others, yet only 7.1% reported LUTS treatment. This finding highlights the large number of women who experience bothersome urinary symptoms without seeking treatment.

The distributions of bladder health using the new BHS/BFI instrument revealed a median global perception of bladder health score of 72 out of 100 (before adjustment for adaptive/coping behaviors) and 55 after adjustment [7]. A downward adjustment in score was made when participants reported using adaptive/coping behaviors such as carrying or wearing incontinence pads, toilet mapping, or staying close to the toilet. When looking only at participants without LUTS (to understand aspects of bladder health that might precede LUTS), scores were higher across all scales and indices, indicating better health, as you would expect. However, scores were not perfect, demonstrating a range of bladder health even among those without LUTS. A notable finding was that 68% of participants in RISE reported using adaptive/coping behaviors related to the bladder, including 40% who reported using pads, liners, and or absorbent underwear, 58% finding the bathrooms everywhere they went (i.e., toilet mapping), and 3% staying as close to a bathroom as possible when away from home. Despite having the best bladder health, 38% of participants in the subset without LUTS also reported using adaptive/coping behaviors, including 11% who reported using pads, 30% toilet mapping, and 2% staying as close to a bathroom as possible when away from home.

Hispanic women had worse bladder health in comparison to non‐Hispanic White women [12]. This appeared to be explained by social determinants of health (SDoH) such as lower levels of education, higher levels of poverty, and no or limited health insurance. Non‐Hispanic Black and Asian women reported better bladder health compared to Non‐Hispanic White women, but these differences were not explained by SDoH. Independent of race, higher socioeconomic position was associated with better bladder health; financial strain and unmet social needs were associated with worse bladder health [13].

Limited knowledge of bladder health and agentic beliefs to prevent or treat LUTS was found, highlighting unmet health education needs [14]. Knowledge and agentic belief scores were associated with social identity and SDoH. Scores were lower in those with Medicaid, less schooling, lower income, transportation instability, food instability, and LUTS.

Women with zero or one chronic health condition reported better bladder health compared to women with multiple chronic health conditions [15]. Women who had begun or had already gone through menopause were around two times more likely to have bladder problems compared with women who hadn′t yet started menopause [16]. Women who told us they avoid toilets when away from home, hold or wait too long to pee, pee before they feel the urge, or strain to pee, also reported more and worse bladder symptoms like having a sudden need to pee, leaking, and pain [17].

It is still early in the data analysis, but so far, RISE has taught us that (1) there is a broad spectrum of bladder health ranging from poor to optimal in US women, (2) there is high utilization of adaptive/coping behaviors (using pads/toilet mapping) even among women without LUTS, and (3) bladder health scores are higher, yet not perfect, in those without LUTS. These findings support the concept that there is more to bladder health than just LUTS and that a novel subclinical population may exist that is optimal for primary LUTS prevention.

## 5 Ongoing Work in RISE FOR HEALTH

The 1‐year follow‐up questionnaire was completed by 2321 of our enrolled participants for a 67.8% retention rate (2321/3422). The 2‐year follow‐up was completed by 2273 participants to date for a 66.4% retention rate (2273/3422) [8]. There are many more findings coming out of RISE from analyses of both the baseline and follow‐up data. The resources developed and the data generated will be made available for use through the NIDDK repository, providing the content for many more accomplishments in the field of bladder health. The PLUS Research Consortium continues to publish RISE data in a wide range of scientific journals across a spectrum of disciplines. In addition, PLUS, in its ongoing engagement with community partners, has produced Common Language Summaries of research findings for the lay public and RISE participants. These summaries are available on the PLUS website as well as on PLUS Research Consortium social media accounts to circulate the message more broadly.

The planned impact of the work of the PLUS Consortium is to lay the foundation for evidence‐based bladder health promotion, enhancing the overall well‐being of girls and women.

## Author Contributions

**Ariana Smith:** conceptualization, writing, reviewing, editing. **Siobahn Sutcliffe:** conceptualization, writing, reviewing, editing.

## Ethics Statement

Studies performed by the PLUS Consortium were IRB approved.

## Consent

Participant consent was obtained for PLUS studies.

## Conflicts of Interest

The authors declare no conflicts of interest.

## Data Availability

The data for the PLUS Consortium is available in the NIDDK repository.
